# Antibiotic approaches to endodontic regeneration

**DOI:** 10.2340/biid.v13.45704

**Published:** 2026-04-22

**Authors:** Samiya Riaz, Asmak Binti Abdul Samat, Muhammad Amber Fareed, Muhammad Sohail Zafar

**Affiliations:** a Fundamental Dental and Medical Sciences, Kulliyyah of Dentistry, International Islamic University Malaysia, Kuantan Campus, 25200, Kuantan, Malaysia; b Institute of Advanced Dental Sciences and Research, Lahore, Pakistan; c Department of Clinical Sciences, College of Dentistry, Ajman University, Ajman, United Arab Emirates; d Centre of Medical and Bio-allied Health Sciences Research, Ajman University, Ajman, United Arab Emirates; e School of Dentistry, Jordan University, Amman, Jordan

**Keywords:** Intracanal medicaments, antibiotics, irrigants, stem cells, regenerative endodontics

## Abstract

Regenerative endodontics (REs) has redefined the management of immature permanent teeth with necrotic pulps by shifting the objective from simple disinfection to biologically driven root maturation and restoration of the dentine–pulp complex. A critical component of this process is the selection of intracanal medicaments that can effectively eliminate microbial infection while preserving the viability of stem cells required for regeneration. This narrative review evaluates the biological and antimicrobial performance of commonly used medicaments, particularly calcium hydroxide (Ca(OH)₂) and triple antibiotic paste (TAP), and discusses their role in RE procedures. A literature search was conducted using PubMed/MEDLINE, Scopus, and Web of Science for articles published between January 2000 and March 2025. Although Ca(OH)₂ remains widely used due to its antimicrobial properties and ability to promote hard tissue formation, concerns remain regarding its high alkalinity and potential adverse effects on stem cell survival. TAP, in contrast, appears to provide a more supportive environment for dental pulp stem cell proliferation and attachment but is associated with drawbacks such as tooth discoloration and antibiotic-related limitations. Emerging alternatives, including double antibiotic paste (DAP) and nano-modified calcium hydroxide formulations, aim to improve the balance between antimicrobial efficacy and biocompatibility. Overall, current evidence emphasizes the importance of optimizing medicament concentration and duration of application to enhance the predictability and long-term success of RE therapy.

**KEY MESSAGES:**

- Calcium hydroxide and antibiotic pastes remain the most widely used intracanal medicaments in REs, each demonstrating distinct advantages and limitations in disinfection efficacy and stem cell compatibility.
- Evidence indicates that medicament concentration plays a critical role in balancing antimicrobial action with the preservation of stem cell viability, which is essential for successful regenerative outcomes.
- Emerging formulations such as nano-calcium hydroxide and modified antibiotic combinations offer promising improvements in biocompatibility and may enhance the predictability of RE procedures.

## Introduction

The increased levels of physical activity among children aged 8 to 12 render them particularly susceptible to traumatic dental injuries, such as molar fractures, dislocations, and avulsions. Infections in the tooth canal system are frequently the result of such injuries [1–4]. Oral trauma is responsible for nearly 48% of all facial injuries [5]. Inflammation, permanent pulpitis, and ultimately pulp necrosis with a loss of vitality are the consequences of untreated damage that affects the neurovascular supply [6]. The progression of chronic carious lesions to pulp necrosis may necessitate extraction. The anatomical vulnerability of the tubercle is also a contributing factor to pulpal necrosis in adolescents, as evidenced by developmental abnormalities such as dens evaginatus [7].

Pulpal necrosis in immature permanent teeth (IPT) with open apices disrupts root maturation as well as apical closure, resulting in weak dentinal walls that elevate the risk of fracture [8]. The ongoing development of roots depends on the preservation of pulpal vitality in IPT [9]. Apexification has consistently been the preferable treatment for teeth of this nature. The treatment aims to eliminate inflammatory signs and symptoms while simultaneously fostering the formation of a calcific barrier by applying calcium hydroxide [Ca(OH)₂], thereby facilitating the final obturation [10].

However, Ca(OH)₂ apexification requires frequent dressings over prolonged periods, which may lead to patient noncompliance and an increased risk of root fracture. Consequently, modern bioactive materials, such as mineral trioxide aggregate (MTA) and tricalcium-silicate cements (Biodentine), have been developed. These materials enhance clinical outcomes and reduce treatment duration; however, they do not promote comprehensive dentine–pulp regeneration or ongoing root formation [11, 12]. Consequently, the long-term prognosis for these teeth is uncertain due to the fragility and susceptibility to fractures of the resulting roots [13].

A fundamental transition from conventional restorative procedures to biological regeneration is represented by stem-cell-based regenerative endodontics (REs). REs has the potential to achieve documented success rates of 90–100% in irreversible pulpitis with necrotic pulps, allowing for authentic tissue regeneration and maturation [14, 15]. RE procedures eradicate infection and promote ongoing root development through apical closure, unlike apexification. The term ‘revascularization’, which was first coined by Banchs [16] emphasizes vascular healing. However, the more comprehensive term ‘maturogenesis’ more accurately reflects the goal of achieving root maturation along with dentine–pulp complex regeneration [17, 18]. Therefore, this narrative review aims to critically evaluate the biological and antimicrobial performance of commonly used intracanal medicaments in REs, with particular emphasis on Ca(OH)_2_, triple antibiotic paste (TAP), and double antibiotic paste (DAP). The literature reviewed was retrieved from PubMed/MEDLINE, Scopus, and Web of Science, covering publications from January 2000 to March 2025.

## Discussion

### Regenerative endodontics and stem cells

REs is founded on the principles of tissue engineering, which consist of three critical components: scaffolds, growth factors, and stem cells [19, 20]. Neural, skeletal, adipogenic, muscular, and dental pulp stem cells (DPSCs) are among the multipotent stem cells that have the capacity to self-renew and differentiate into several lineages [21]. Their fundamental role in the regeneration of dental tissue stems from their capacity for continuous division and repair [22]. The repair of the dentine-pulp complex can be facilitated by inducing odontoblast-like cell development from stem cells when combined with biocompatible scaffolds and appropriate growth factors, such as transforming growth factor-beta (TGF-β) [23].

In regenerative endodontics, DPSCs are essential due to their ability to form odontoblast-like morphologies [24, 25]. At the sites of pulpal injury, these cells release dentine matrix [26, 27] In the event of pulpal injury, the radicular dentine’s DPSCs become metabolically active and differentiate into odontoblasts to initiate the dentine–pulp complex repair. The multilineage capacity of DPSCs to differentiate into neural progenitors, myogenic, chondrocytic, odontogenic, odontoblastic, and adipogenic cell types was first demonstrated by Gronthos et al. in human third molars [28, 29]. Their therapeutic potential is underscored by their superior regenerative capacity compared with alternative stem cell sources [30,–32]. The efficacy of RE therapy depends on the milieu surrounding the cells and the intrinsic characteristics of stem cells. The apical portion of necrotic pulps may contain viable DPSCs, particularly in juvenile molars [20, 33]. The objective of regenerative procedures is to create an environment that is conducive to the proliferation and differentiation of residual cells. This procedure requires efficient, physiologically compatible disinfection of the root canal system, which involves reducing or removing instrumentation to preserve the integrity of periapical tissue and dentinal walls [34, 35].

The results of REs are significantly affected by the disinfection process. Stem cell viability must be preserved while the selected irrigants and intracanal medicaments exhibit bactericidal efficacy [36, 37]. It is imperative to achieve this equilibrium; however, excessive chemical aggression may damage remnant stem cells and impede tissue regeneration [19], while necrotic detritus and microbial pollutants must be completely eliminated [38, 39]. The dentine–pulp complex’s healing, revascularization, and subsequent apical closure are all facilitated by the favorable chemical and biological conditions in the canal.

To preserve stem cell viability, recent research has focused on reducing the concentration of disinfectants used in regenerative treatments [40, 41]. The objective of these initiatives is to cultivate an environment that is ‘stem-cell-friendly’ and encourages healing by reducing cytotoxicity and preserving antimicrobial efficacy. Although numerous studies have examined the antibacterial effects of mixed irrigants and medicaments [42–45], there are limited studies on the synergistic effect of these agents on the adhesion and proliferation of DPSCs on dentine [10, 36, 46, 47].

### Intracanal medicaments and their effects

The proper execution of RE procedures depends on the efficient chemical cleaning and disinfection of the canal system. The effective eradication of bacteria, the extraction of necrotic debris, and the creation of an environment favorable for stem cell adhesion and proliferation necessitate the careful selection and sequencing of irrigants and intracanal medicaments. The smear layer on dentin, measuring around 2–5 μm in thickness, can impede stem cell adhesion [48]. Therefore, the irrigants are essential for its removal and for exposing dentinal tubules to promote cell adhesion.

The preferred irrigant in modern endodontics is sodium hypochlorite (NaOCl), used at concentrations of 0.5% to 6%, with dual functions of dissolving organic tissues and disinfecting the canal [49]. However, NaOCl alone cannot eradicate the inorganic component of the stain layer. Chelating drugs, such as ethylenediaminetetraacetic acid (EDTA, typically 15–17%), are used to mitigate this limitation [50]. EDTA has been identified as the most compatible irrigant for stem cells and is recommended as a final rinse in regeneration protocols to enhance stem cell adhesion, proliferation, and differentiation [49]. Furthermore, EDTA mitigates the cytotoxic effects of NaOCl, therefore promoting the reestablishment of a biologically conducive canal environment [51]. Research has shown that the sequential irrigation of NaOCl and EDTA, even in the absence of direct combination, can result in chemical interactions that produce hazardous by-products harmful to stem cell viability [52]. This observation underscores the need of following exact irrigation regimens during REPs.

The most frequently employed intracanal medications are Ca(OH)₂ and TAP. The antibacterial efficacy of TAP, which contains metronidazole, ciprofloxacin, and minocycline, is broad. The aesthetics of both crowns and roots have been influenced by the minocycline component, which has been linked to tooth discoloration [53]. To overcome the discoloration associated with minocycline, a modified formulation known as DAP was introduced. DAP consists of metronidazole and ciprofloxacin, excluding minocycline, and was developed to reduce esthetic complications while maintaining antimicrobial efficacy; however, DAP has been documented to exhibit cytotoxic effects on stem cells, despite this alteration [54].

The viability and differentiation of stem cells, specifically human DPSCs [10, 36, 55, 56], stem cells of the apical papilla (SCAP) [57], and stem cells from human exfoliated deciduous teeth (SHED) [58], have been the subject of numerous studies. Numerous studies have demonstrated the cytotoxicity of Ca(OH)₂ at elevated pH levels, despite its long-standing use as an intracanal medicament for RE procedures [16, 46, 59]. In contrast, Althumairy et al. demonstrated that stem cell viability is significantly influenced by TAP and DAP levels [57]. The viability of stem cells is significantly reduced by both medications at elevated concentrations (1 g/ml), whereas the beneficial effects are maintained at reduced concentrations (1 mg/ml). Additionally, stem cell proliferation is stimulated by Ca(OH)₂ at suitable dilutions. Additionally, Labban et al. proposed that stem cell viability and proliferation are optimally facilitated by reduced concentrations of TAP, DAP, and Ca(OH)₂ (0.3–2.5 mg/ml) [55].

Alghilan et al. have reported that the therapeutic dosages of these medications, which are extensively used in clinical settings, are inadequately characterized, despite the fact that they optimize antibacterial effectiveness while minimizing cytotoxicity [46]. Numerous in vitro studies assess the direct effects of pharmaceuticals by directly exposing stem cells to the substances [60–62]. However, these methodologies may not accurately reflect clinical scenarios in which pharmaceuticals exert indirect effects via dentinal diffusion. It is imperative to understand this indirect relationship to optimize RE procedure outcomes.

The efficacy of intracanal medicaments has been further supported by recent investigations. Riaz et al. demonstrated that the viability and attachment of DPSCs are improved in dentine treated with TAP in comparison to Ca(OH)₂, indicating a more favorable microenvironment for stem cell survival [36]. The synergistic effect of combining Ca(OH)₂ with chlorhexidine (CHX) to enhance periapical healing was the primary focus of their previous clinical research. Similarly, Hafez et al. demonstrated that nano-calcium hydroxide formulations potentially address the cytotoxic limits of traditional formulations by enhancing stem cell viability and differentiation [63]. Farjaminejad et al. conducted a comprehensive evaluation of RE procedures, concluding that both Ca(OH)₂ and TAP are clinically efficacious for stem-cell-mediated regeneration and root growth [23]. Similar results were confirmed by concurrent analyses, underscoring the importance of balancing disinfection efficacy with stem cell preservation [64, 65]. Maru et al. reaffirmed the ongoing significance of Ca(OH)₂ as a preferred medicament in pediatric endodontics [66], while another study confirmed that both TAP and Ca(OH)₂ effectively eliminate polymicrobial biofilms, creating conducive environments for stem cell adhesion and survival [67].

Recent research consistently establishes Ca(OH)_2_ as a dependable intracanal medicament. In a randomized clinical experiment with a 36-month follow-up, non-setting Ca(OH)_2_ shown comparable efficacy to modified TAP in regenerative treatments, showing no significant differences in success or survival rates [68]. Although this study had limitations such as lack of blinding and only including incisors, the long-term follow-up is valuable. Likewise, no notable differences in postoperative pain were detected among Ca(OH)_2_, CHX, or their combination in retreatment scenarios, indicating that the selection of medicament may not significantly affect short-term discomfort when protocols are standardized [69]. However, the subjective nature of pain rating must be taken into account when interpreting these data.

From an antibacterial standpoint, probiotics exhibited effectiveness akin to TAP in diminishing *E. faecalis*, generating interest in biologically safer options due to apprehensions around antibiotic resistance and cytotoxicity [70]. However, the short-term microbiological focus restricts inferences regarding long-term clinical results.

In vitro studies indicated improved antimicrobial penetration with nanoparticle-modified formulations and emphasized the impact of medications on sealer penetration [71, 72]. Although intriguing, these laboratory results necessitate careful interpretation owing to their restricted clinical relevance. Observational data further demonstrate diversity in healing results among medications, underscoring the necessity for more rigorously planned comparative studies [73].

Table 1 summarizes the current evidence on intracanal medicaments used in REs, highlighting study design, level of evidence, and methodological limitations to provide a concise overview of the existing literature. The level of evidence and risk of bias were assessed according to the Oxford Centre for Evidence-Based Medicine (OCEBM) 2011 criteria, with Level I representing the highest level of evidence and Level V the lowest [74]. While intracanal medicaments are crucial for RE procedures, the research presents contrasting clinical and biological results, probably due to differences in medicament type, concentration, application duration, and treatment protocols. Although antibiotic-based pastes demonstrate significant antibacterial effectiveness, their potential cytotoxicity to stem cells and inconsistent long-term therapeutic superiority compared to Ca(OH)_2_ continue to be contentious issues. Ca(OH)_2_ presents practical benefits in cost, availability, and use, especially in resource-limited environments, while issues related to antimicrobial resistance and accessibility restrict the broad implementation of antibiotic formulations. Thus, the selection of medications must reconcile antibacterial efficacy with biological compatibility, cost-effectiveness, and practical clinical limitations.

**Table 1:** Intracanal medicaments in regenerative endodontics: study characteristics, level of evidence, and risk of bias.

	Author(s)/year	Study type	Medicaments studied	Experimental model/population	Key findings	Level of evidence*	Main limitations/risk of bias*
1	Althumairy et al., 2014	In vitro	Ca(OH)₂, TAP, DAP	SCAP	High concentrations of TAP/DAP reduced viability; low concentrations and Ca(OH)₂ promoted proliferation.	Level V	In vitro model; no clinical correlation
2	Labban et al., 2014	In vitro	Ca(OH)₂, TAP, DAP	DPSCs	Low concentrations favored stem cell viability.	Level V	Short-term culture; no in vivo validation
3	Sabrah et al., 2015	In vitro	DAP, TAP	DPSCs	DAP was more cytotoxic; TAP better preserved viability.	Level V	Cell culture only; discoloration assessed indirectly
4	Alghilan et al., 2016	In vitro	Ca(OH)₂, TAP	DPSCs on dentine	TAP enhanced attachment and proliferation.	Level V	Artificial dentine disc model
5	Ruparel et al., 2012	In vitro	TAP, Ca(OH)₂	DPSCs (direct contact)	High-dose TAP reduced viability.	Level V	Direct-contact exaggerates cytotoxicity
6	Riaz et al., 2022	In vitro	TAP vs. Ca(OH)₂	DPSCs on dentine	TAP-treated dentine supported higher viability.	Level V	Single experimental model
7	Riaz et al., 2018	Clinical	Ca(OH)₂ + CHX	Immature necrotic teeth	Improved periapical healing and disinfection.	Level III	Non-randomized; small sample
8	Maru et al., 2022	Clinical	Ca(OH)₂	Pediatric RE procedures	Ca(OH)₂ effective as intracanal medicament.	Level III–IV	Limited follow-up
9	Hafez et al., 2024	In vitro	Nano-Ca(OH)₂	DPSCs	Nano-Ca(OH)₂ improved viability and differentiation.	Level V	Experimental formulation
10	Ibrahim et al., 2025	In vitro	Ca(OH)₂, TAP	Polymicrobial biofilm	Both medicaments eradicated biofilms.	Level V	Short-term lab model
11	Farjaminejad et al., 2025	Systematic review	Ca(OH)₂, TAP	Clinical RE procedures	Comparable clinical efficacy.	Level I	Heterogeneity among studies
12	Manoharan et al., 2025	Systematic review	Ca(OH)₂, antibiotic pastes	Multiple studies	Balance between disinfection and biocompatibility.	Level I	Narrative synthesis bias
13	Aga et al., 2025	Narrative review	Ca(OH)₂, TAP, DAP	Literature	Effectiveness concentration-dependent.	Level V	Non-systematic review
14	Al-Qudah et al., 2023	Randomized clinical trial	Non-setting Ca(OH)₂ vs. Modified TAP	Nonvital immature permanent teeth (RE procedures)	Both medicaments had high success and survival at 36 mo; no significant difference.	Level II (RCT)	Moderate risk: clinical outcome measures not blinded; limited to anterior teeth.
15	Angın et al., 2024	Randomized clinical trial	Ca(OH)₂, CHX gel, Ca(OH)₂ + CHX	Single-root retreatment teeth, PTAP	No significant differences in postoperative pain/flare among groups.	Level II (RCT)	Moderate risk: subjective pain outcomes; potential placebo effect.
16	Dixit et al., 2024	In vivo clinical	CH, TAP, PBs	Young permanent teeth (12–17 y)	All reduced E. faecalis; PBs had similar efficacy to TAP.	Level III (Clinical in vivo)	Moderate risk: small sample; short-term microbiological endpoints.
17	Kaukab & Nekkanti 2025	In vitro	Ca(OH)₂ + nano-Ca(OH)₂; ZnO + nano-ZnO; controls	Extracted primary teeth	Nanoparticles enhanced antimicrobial efficacy and penetration.	Level V (In vitro)	Laboratory model; clinical relevance unknown.
18	Bayram & Bayram 2025	In vitro	Common intracanal medicaments	Dentine/root canals (confocal study)	Medicaments influenced dentinal tubule penetration of sealers.	Level V (In vitro)	In vitro: may not reflect clinical conditions.
19	Chethan et al., 2024	Narrative/clinical case series	Multiple medicaments	Apical periodontitis patients	Medicaments associated with healing variations; need more evidence.	Level IV (Observational/Case series)	Lack of controls; narrative outcomes.

* OCEBM criteria were used to assess level of evidence and risk of bias.

Ca(OH)₂: Calcium hydroxide; TAP: Triple antibiotic paster; DAP: Double antibiotic paste; CHX: Chlorhexidine; SCAP: DPSCs Dental pulp stem cells.

### Clinical recommendations

Based on available clinical and experimental evidence, low-concentration antibiotic pastes are generally preferred in RE procedures, with recommended concentrations not exceeding 0.1–1 mg/mL to minimize cytotoxic effects on stem cells of the apical papilla. DAP, a modified formulation of TAP without minocycline, may be favored over TAP when concerns regarding tooth discoloration exists, particularly in anterior teeth. Ca(OH)_2_remains an effective intracanal medicament, with clinical protocols commonly limiting its intracanal duration to short-term application (approximately 1–4 weeks) to reduce potential adverse effects on dentin integrity and regenerative outcomes. Strategies to minimize discoloration include avoiding minocycline-containing formulations, sealing coronal dentin with bonding agents, and preferential use of Ca(OH)_2_ in esthetically sensitive cases. In infected immature teeth with minimal esthetic concern, antibiotic pastes may be considered for enhanced antimicrobial control, whereas Ca(OH)_2_ may be preferable in cases prioritizing cost, availability, and use in low-resource clinical settings.

## Conclusion

In REs, intracanal medications eliminate bacteria and create a biologically favorable environment for tissue regeneration, differentiation, and stem cell survival. The switch from traditional apexification to REs marks a major step toward biological regeneration of the dentine-pulp complex. Common drugs include Ca(OH)₂ and TAP. Each has pros and cons. Ca(OH)₂ can be toxic at high concentrations, despite its antibacterial properties and its role in hard tissue growth. TAP enhances stem cell adhesion and vitality but may cause antimicrobial resistance and coloring. Novel approaches, such as nano-calcium hydroxide compositions and DAP, may balance biological compatibility and disinfection efficacy. Future regeneration therapy research should focus on drug doses and exposure durations to improve clinical outcomes. Research on novel bioactive chemicals, nanocarrier systems, and stem-cell-compatible irrigant methods is needed to improve therapeutic methods. Next-generation RE procedures will succeed if they combine scaffold-assisted regeneration, cellular preservation, and controlled disinfection.

## Data Availability

Data sharing is not applicable to this article, as no new data were created or analyzed in this study.
